# Early vascular aging as an index of cardiovascular risk in healthy adults: confirmatory factor analysis from the EVasCu study

**DOI:** 10.1186/s12933-023-01947-9

**Published:** 2023-08-17

**Authors:** Alicia Saz-Lara, Iván Cavero-Redondo, Carlos Pascual-Morena, Irene Martínez-García, Eva Rodríguez-Gutiérrez, Maribel Lucerón-Lucas-Torres, Bruno Bizzozero-Peroni, Nerea Moreno-Herráiz, Arturo Martínez-Rodrigo

**Affiliations:** 1 Health and Social Research Center, University of Castilla-La Mancha, Cuenca, Spain; 2 Universidad Autónoma de Chile, Facultad de Ciencias de la Salud, Talca, Chile; 3 Instituto Superior de Educación Física, Universidad de la Republica, 40000 Rivera, Uruguay; 4 Research Group in Electronic, Biomedical, and Telecommunication Engineering, University of Castilla-La Mancha, Cuenca, Spain

**Keywords:** Early vascular aging, Pulse pressure, Glycated hemoglobin, Pulse wave velocity, Advanced glycation end products, Healthy adults

## Abstract

**Background:**

The concept of early vascular aging (EVA) represents a potentially beneficial model for future research into the pathophysiological mechanisms underlying the early manifestations of cardiovascular disease. For this reason, the aims of this study were to verify by confirmatory factor analysis the concept of EVA on a single factor based on vascular, clinical and biochemical parameters in a healthy adult population and to develop a statistical model to estimate the EVA index from variables collected in a dataset to classify patients into different cardiovascular risk groups: healthy vascular aging (HVA) and EVA.

**Methods:**

The EVasCu study, a cross-sectional study, was based on data obtained from 390 healthy adults. To examine the construct validity of a single-factor model to measure accelerated vascular aging, different models including vascular, clinical and biochemical parameters were examined. In addition, unsupervised clustering techniques (using both K-means and hierarchical methods) were used to identify groups of patients sharing similar characteristics in terms of the analysed variables to classify patients into different cardiovascular risk groups: HVA and EVA.

**Results:**

Our data show that a single-factor model including pulse pressure, glycated hemoglobin A1c, pulse wave velocity and advanced glycation end products shows the best construct validity for the EVA index*.* The optimal value of the risk groups to separate patients is K = 2 (HVA and EVA).

**Conclusions:**

The EVA index proved to be an adequate model to classify patients into different cardiovascular risk groups, which could be valuable in guiding future preventive and therapeutic interventions.

**Supplementary Information:**

The online version contains supplementary material available at 10.1186/s12933-023-01947-9.

## Introduction

One hypothesis to explain some aspects of early cardiovascular disease (CVD) is that of early vascular ageing (EVA) as part of a broader process of early biological ageing [1, 2]. This could be the case for individuals with classical risk factors or with a strong family history of early manifestations of CVD [1, 3]. Healthy vascular aging (HVA) is associated with a gradual change in vascular structure and function, leading to a decrease in arterial distensibility and an increase in arterial stiffness [4, 5]. This is influenced by age-dependent structural and biochemical changes, e.g., in the elastin and collagen content of the vascular wall. Superimposed on this physiological process is EVA, which is accompanied by characteristic lesions and plaque formation and begins with an increase in the thickness of the intima media in the vessel wall [4–6].

Systems that assess traditional risk factors greatly improve risk prediction but classify only a small proportion of asymptomatic individuals over the age of 40 as high risk, which is contradictory when observing the high CVD morbidity and mortality at this age [7]. This discrepancy is known as the detection gap, and it can be argued that current screening and diagnostic methods are insufficient to identify those at risk of developing a cardiovascular event [8]. In calculating risk, the vast majority do not consider emerging risk factors, and they are likely to exert a greater influence on overall individual risk than traditional or classical risk factors [9].

Based on the available evidence, the EVA concept represents a potentially effective model for future research into the pathophysiological mechanisms underlying the early manifestations of CVD [1, 3]. Some studies support the association of different parameters related to vascular aging, such as arterial stiffness, endothelial dysfunction and carotid intima-media thickness [2, 5]. In addition, they propose the EVA index as a predictor of some chronic pathologies such as cancer, diabetes mellitus or CVD in subjects at increased cardiovascular risk [1]. Other findings show that these emerging markers could be more predictive than classical markers, showing additional better prediction when combined with classical parameters of cardiovascular risk [9].

Since it has been recognized that there is a discrepancy between a patient's chronological age and their signs and symptoms of vascular age and that in some individuals, the vascular aging process is more accelerated than in others, increasing the risk of premature manifestations of CVD [3], the aims of this study were (1) to verify by confirmatory factor analysis the concept of single-factor EVA based on vascular, clinical and biochemical parameters in a healthy adult population and according to sex and (2) to develop a statistical model to estimate the EVA index from the variables collected in a dataset to classify patients into two different cardiovascular risk groups: HVA and EVA.

## Methods

### Design, participants, and sample size

The EVasCu study, a cross-sectional design study, is based on data obtained from healthy adult subjects from the city of Cuenca, Spain (collected from June to December 2022). The inclusion and exclusion criteria for participants are shown in Additional file 1: Table S1. This study was conducted according to the guidelines for reporting observational studies “Strengthening the Reporting of Observational Studies in Epidemiology (STROBE) Statement” [10].

The sample size was calculated using Epidat software and indicated that 355 participants would provide an estimated effect size of 1, with an alpha risk of 0.05 and an absolute precision level of 0.04 to detect a statistically significant result for the EVA index [11]. Subjects meeting the inclusion and exclusion criteria were invited to participate in the study, and eventually, 390 participants were enrolled.

### Ethical considerations

The research protocol of this study was approved by the Clinical Research Ethics Committee of the Cuenca Health Area (REG: 2022/PI2022). Written informed consent to participate was obtained from all subjects included in the study. All procedures performed in this study were in accordance with the Declaration of Helsinki and its later amendments or comparable ethical standards for experiments involving humans [12].

### Variables

#### Vascular parameters

Arterial stiffness was measured using oscillometric techniques with Mobil-O-Graph® (IEM GmbH) and VaSera (FUKUDA-DENSHI). Mobil-O-Graph® measures aortic pulse wave velocity (a-PWv) and augmentation index (AIx75), which were calculated as the mean of two repeated measurements, separated by 5 min each, while VaSera measures the cardio-ankle vascular index (CAVI). These parameters were measured in a quiet place and after a 5-min rest period using cuff size according to the participant’s arm/s and/or lower limb circumference.

Mean and maximal intima-media thickness (IMT) was measured by ultrasound with the Sonosite SII device (Sonosite Inc., Bothell, Washington, USA). IMT was calculated as the mean measurement of the right and left carotid arteries.

#### Clinical parameters

Pulse pressure (PP) was obtained from the difference between mean systolic blood pressure (SBP) and diastolic blood pressure (DBP). Blood pressure was measured in a quiet place and after a 5-min rest period using the Omron® M5-I monitor (Omron Healthcare UK Ltd. with a cuff size according to the participant’s arm circumference. SBP and DBP were calculated as the mean of two repeated measurements, separated by 5 min each.

Advanced glycation end products (AGEs) were measured by skin autofluorescence (SAF) with the AGE Reader® device. AGEs were calculated as the mean of the measurements from both arms. The mean for each arm was calculated as the mean of three repeated measurements.

#### Biochemical parameters

Glucose and ultrasensitive C-reactive protein (CRP) determinations were measured on a Roche Diagnostics® Cobas 8000 system, and insulin determinations were measured on the Abbott ® Architect platform. Glycated hemoglobin A1c (HbA1c) was determined by high-performance liquid chromatography using the ADAMS A1c HA-8180V analyser from A. Menarini Diagnostics®. Samples were collected between 8 a.m. and 9 a.m. and after 12 h of fasting.

### Statistical analysis

#### Confirmatory factor analysis

To examine the construct validity of a single-factor model to measure the EVA index, different models including vascular, clinical, and biochemical parameters were examined to determine which variables from these three groups showed the best fit. Regression coefficients > 0.3 and a statistical significance of p < 0.05 were considered criteria for including a variable in the EVA construct. For single-factor construct validity models, a model was considered to have a good fit if the comparative fit index (CFI) was > 0.96 and the root mean square residual (SRMR) was < 0.008 [13].

#### Choosing the optimal number of risk groups

The optimal number of risk groups (K) was determined considering the nature of the variables that best formed the construct validity of the EVA index. To accomplish this task, different values of K, ranging from 2 to 5, were explored and used in different algorithms to determine the optimal selection of groups in the dataset. First, the Calinski‒Harabasz [14] and Davies‒Bouldin [15] indices, which assess the relationship between the dispersion within the groups themselves and the dispersion between them, were calculated. A higher value indicates better separation between groups and less dispersion within each group in the Calinski‒Harabasz algorithm, while a lower value in the Davies‒Bouldin algorithm indicates better separation between groups and greater cohesion within each group.

Second, the silhouette index, which computes the mean distance of each observation to the observations in its own group (cohesion) and the mean distance to the observations in the other groups (separation) [16], was also calculated. The silhouette index varies between − 1 and 1, where a value close to 1 suggests that the observations within a group are very close to each other (cohesion) and far from the observations of other groups (separation).

#### Cluster calculation and validation

To calculate the assignment of each patient to each of the vascular aging risk groups (K), two different unsupervised clustering methodologies were computed. First, the K-means algorithm was implemented, which assigns each patient to the closest group or centroid, considering that centroids are representative points of each group in the multidimensional space and correspond to the mean of all points in that group [17]. This process was repeated up to a maximum of 100 times or until the relative changes in centroid positions were negligible. Second, hierarchical clustering was also used to assign subjects to each vascular aging risk group based on the four variables selected in the construct. Hierarchical clustering is an unsupervised approach that constructs a tree structure (dendrogram), where each level represents a partition of the data into different groups [18] (Additional file 1: Fig. S1). Finally, the concordance index was calculated as the ratio of the total number of subjects assigned to the same group by both methodologies.

Furthermore, to verify the similarity in the assignment of subjects to different groups with these two methodologies, the adjusted Rand index (ARI) was calculated [19]. The ARI is a measure of similarity between two partitions of a dataset and compares the agreement between group assignments in two different clustering methodologies. The ARI ranges from − 1 to 1, where a value of 1 indicates a perfect match between group assignments, while a value of 0 indicates that the assignments match to the same extent by chance.

#### Dimension reduction

The multivariable approach to the construct proposed in this study makes it difficult to visually analyse the dispersion of observations and their respective group assignments. To address this issue, dimensionality reduction was performed using principal component analysis (PCA) [20]. PCA allows the original variables to be transformed into orthogonal components that preserve as much variance as possible. This enables visual exploration and detection of patterns and trends through scatter plots of the subjects, facilitating the assessment of group clarity and separation and providing insight into the importance of each variable in forming the principal components.

All abovementioned analyses were also performed by sex. Statistical analyses were performed using STATA 15 and MATLAB 2022b.

## Results

### Characteristics of study participants

The EVasCu study sample included a total of 390 participants, of whom 246 (63.1%) were women. The mean age of the participants was 42.0 ± 13.1 years. Table 1 shows the baseline characteristics of the enrolled population.

**Table 1:** Characteristics of the EVasCu population included in this analysis

Variables	Total *n* = 390	Men *n* = 144	Women *n* = 246
Age (years)	42.0 ± 13.1	42.3 ± 12.5	41.8 ± 13.5
Current smokers (%)	12.3	9.8	13.8
BMI (kg/m^2^)	24.9 ± 4.3	25.7 ± 3.5	24.4 ± 4.5
Body fat (%)	27.2 ± 9.4	19.9 ± 6.9	31.5 ± 7.8
Waist circumference (cm)	82.7 ± 12.8	88.6 ± 11.4	79.1 ± 12.3
SBP (mmHg)	116.7 ± 15.2	125.1 ± 12.8	111.8 ± 14.4
DBP (mmHg)	70.4 ± 10.6	72.3 ± 10.3	69.2 ± 10.6
PP (mmHg)	46.3 ± 9.9	52.8 ± 8.7	42.5 ± 8.5
PCR (mg/L)	1.8 ± 4.1	1.5 ± 2.9	2.0 ± 4.6
Glucose (mg/dL)	89.4 ± 9.9	91.5 ± 9.8	88.2 ± 9.8
Insulin	8.5 ± 6.1	8.3 ± 5.4	8.7 ± 6.4
HbA1c (%)	5.2 ± 0.3	5.2 ± 0.4	5.2 ± 0.3
PWv (m/s)	6.3 ± 1.4	6.5 ± 1.3	6.2 ± 1.4
AIx75 (%)	16.7 ± 12.0	10.0 ± 10.1	20.7 ± 10.9
CAVI (m/s)	7.1 ± 1.2	7.2 ± 1.3	7.0 ± 1.1
Median-IMT (mm)	0.2 ± 0.1	0.2 ± 0.1	0.2 ± 0.1
Maximum-IMT (mm)	0.5 ± 0.1	0.5 ± 0.2	0.4 ± 0.1
AGEs (AU)	1.9 ± 0.4	1.9 ± 0.5	1.9 ± 0.4

Values are presented in mean ± SD

*AGEs* advanced glycation end products, *AIx* augmentation index, *BMI* body mass index, *CAVI* cardio-ankle vascular index, *DBP* diastolic blood pressure, *HbA1c* glycated hemoglobin A1c, *IMT* intima media thickness, *PCR* C-reactive protein, *PP* pulse pressure, *PWv* pulse wave velocity, *SBP* systolic blood pressure

### EVA as an index of cardiovascular risk

**Fig. 1:**
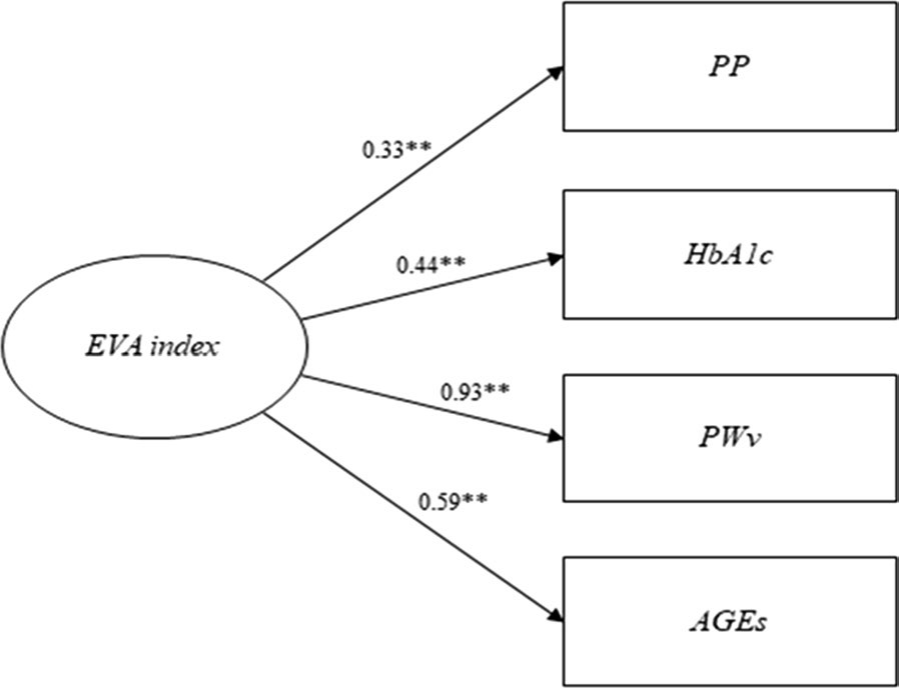
Factor loading and goodness-of-fit indexes of single-factor models for EVA index

Figure 1 shows the single-factor model proposed for the analysis of the factor structure of the EVA index. The EVA index model with the best goodness-of-fit was the model that included PP, HbA1c, PWv and AGEs [Chi^2^ (df) = 4.26 (2), p = 0.119, CFI = 0.991, SRMR = 0.026]. The overall estimates of the factor loadings were 0.33, 0.44, 0.93, and 0.59 for PP, HbA1c, PWv and AGEs, respectively. Similar results were obtained when the single-factor models were estimated to compare goodness-of-fit by sex (Additional file 1: Figs. S2 and S3).

### Optimal number of groups to be analysed

Table 2 shows the results obtained for the Davies‒Bouldin (0.9078) and Calinski‒Harabasz (316.27) algorithms when different values of K were used. Both methods agree that the optimal value of vascular aging risk groups to separate the subjects is K = 2 (HVA and EVA). As can be observed from the silhouette index, K = 2 was also the best result obtained with this algorithm (0.5972).

**Table 2:** Results for the optimal number of vascular aging risk groups

	K = 2	K = 3	K = 4	K = 5
DaviesBoulding	**0.9078**	0.9646	0.9558	0.9372
Calinski–Harabasz	**316.27**	290.63	279.42	275.20
Silhouette index	**0.5972**	0.5342	0.5401	0.5039

### Cluster analysis

The relationship between subject assignments to the two groups (HVA and EVA) using both clustering methods, K-means and hierarchical, is shown in Additional file 1: Table S2. The HVA of the K-means method is composed of 224 subjects who are also in the HVA of the hierarchical method, together with 15 additional subjects assigned to EVA. Furthermore, no subjects found in the HVA of the hierarchical method are assigned to the EVA of the K-means method, but 142 subjects are assigned to EVA of both methods. The concordance index was 0.961. Similarly, the ARI was 0.8481, which confirms that there is good concordance between the group assignments made by the two clustering methods and indicates that the identified groups are quite robust and consistent across different clustering approaches.

Furthermore, the same K-means clustering analysis was performed for K = 2 (HVA and EVA), considering sex stratification. No significant differences were found in the groupings, obtaining visually homologous separations between strata. In this regard, the silhouette index and the mean distance of all the observations to their respective centroids for both strata were calculated, obtaining very similar results, as shown in Additional file 1: Table S3.

### Visual representation of clusters

**Fig. 2:**
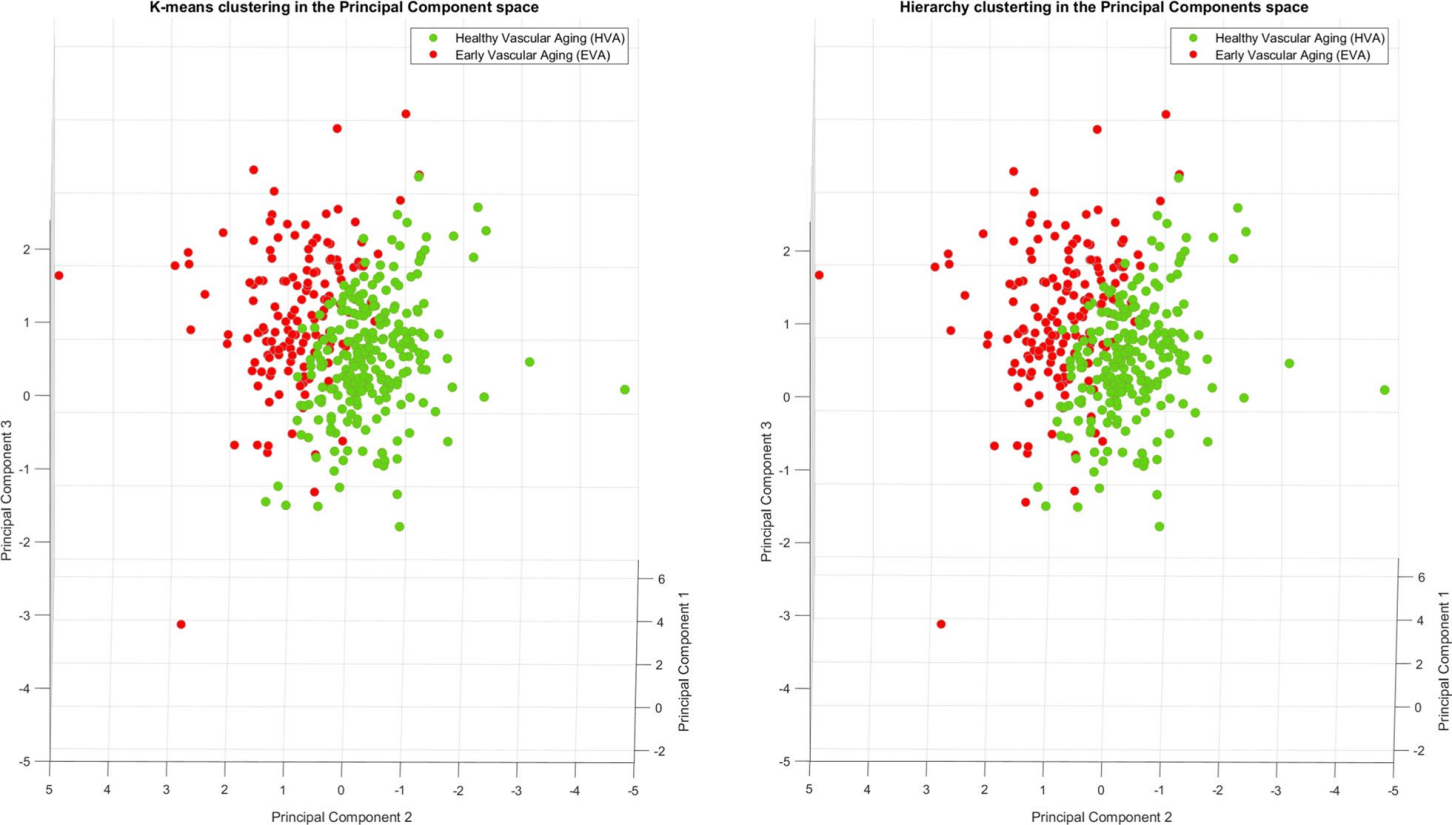
Visual representation of the assignment in the two groups (HVA and EVA) in a three-dimensional space using K-means and hierarchical clustering methods

After PCA was applied to the EVA index model variables (PP, HbA1c, PWv and AGEs), four principal components were obtained, explaining 48.5%, 24%, 17.2%, and 10.3% of the total variance, respectively. While the sum of the first two components explained only 72.5% of the total variance, including a third component increased this percentage to 89.7%. Figure 2 shows the dispersion of the subjects in a three-dimensional space and their assignment in the two groups (HVA and EVA) for both K-means and hierarchical clustering methods. The assignment models obtained by both methodologies are very similar. This result reflects that both clustering methods have identified similar patterns in the data, which may indicate that the resulting clustering is consistent and represents the underlying structure of the data. When this analysis was conducted by sex, similar results were found (Additional file 1: Figs. S4 and S5).

**Fig. 3:**
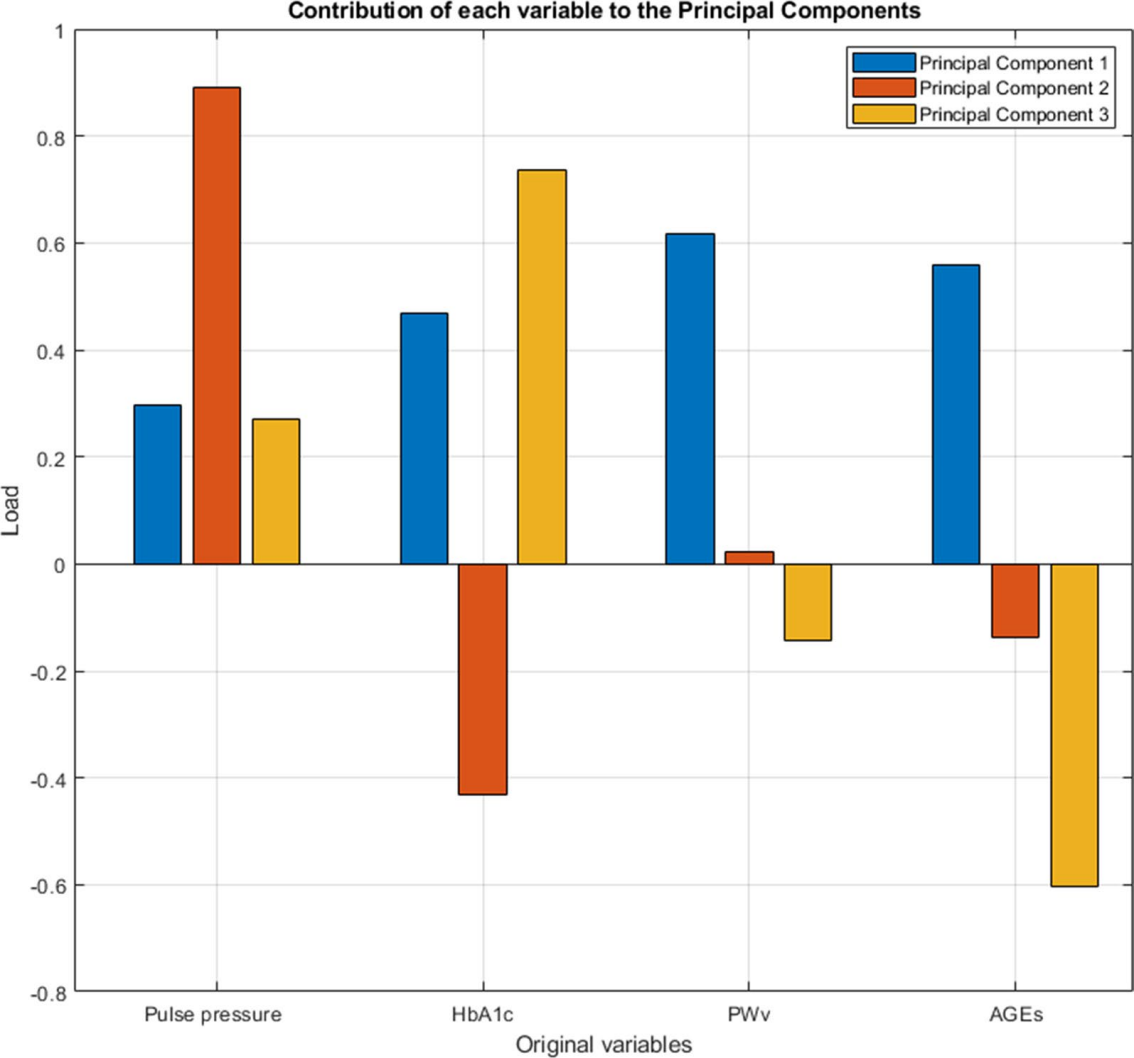
Contribution of each variable of the EVA index model to each principal component

Finally, Fig. 3 shows the contribution of each variable of the EVA index model (PP, HbA1c, PWv and AGEs) to the formation of the first three principal components. Additional file 1: Figs. S6 and S7 show the contribution of each EVA model variable by sex.

## Discussion

Currently, knowing which parameters are associated with the EVA index can help assess an individual’s risk of developing the condition. This may be useful in developing preventive strategies for those at increased risk. In addition, differentiating individuals with HVA from those with EVA can be challenging, as the transition from a healthy state to vascular aging is a gradual process and can be influenced by several factors, such as age, lifestyle, genetics and comorbidities. Our data, using confirmatory factor analysis, show that a single-factor model including PP, PWv, HbA1c and AGEs shows the best construct validity for the EVA index. Furthermore, our findings show that the combination of these four parameters allows the creation of a cohesive and robust clustering model that could be used to classify new cases of HVA and EVA.

Overall, the identification of relevant parameters associated with the EVA index is crucial for early detection, risk assessment, treatment monitoring and research. We found that the factors that best collectively explain the EVA index in this study, which includes a theoretically healthy population without a clinical diagnosis of any disease, are PP, PWv, HbA1c, and AGEs. However, it is important to note that these parameters should not be considered in isolation, and a comprehensive assessment of an individual’s clinical and medical history should also be considered when making a diagnosis of the EVA index.

Commonly higher PP, PWv, HbA1c and AGEs have been independently associated with an increased EVA index, and all these parameters are strong predictors of CVD and mortality [21, 22], in the case of HbA1c even in the nondiabetic population [23]. The pathophysiological mechanisms by which each of these parameters is associated with the EVA index are as follows: (1) as arteries stiffen, they lose their ability to buffer the pressure changes generated by the heartbeat, leading to an increase in PP [24]; (2) with vascular aging, there is a gradual accumulation of structural changes in the arterial wall, including an increase in collagen content and cross-linking, a decrease in elastin content, and the development of vascular calcification. These changes lead to a loss of arterial elasticity and compliance, making the arterial walls stiffer and less able to respond to changes in blood pressure [25]; (3) elevated blood glucose levels can lead to damage to the blood vessels, including the thickening of the arterial walls, decreased elasticity of the blood vessels, and increased risk of plaque formation [26]; and (4) SAF is a noninvasive technique that measures the accumulation of AGEs in the skin [27]. AGEs can activate inflammatory pathways in the endothelial cells that line the inner walls of blood vessels, leading to the recruitment of immune cells and further inflammation, oxidative stress, and damage to the blood vessel walls [28].

Correctly classifying HVA and EVA is crucial for several reasons: to help with early detection and intervention, which can prevent or delay the onset of CVD [29]; to ensure that individuals receive the appropriate treatment based on their vascular condition, which can improve treatment outcomes; and to help in assessing their risk of developing CVD. This information can be useful in developing preventive strategies and lifestyle modifications to reduce their risk and can also be useful in public health efforts to reduce the burden of CVD, providing insights into the risk factors and mechanisms of the disease [1]. Finally, studying the underlying mechanisms of early vascular aging can help in the development of new therapies and interventions [30].

Our results should be interpreted with caution, as they come from a cross-sectional study and, therefore, do not establish a temporal relationship between the parameters included and the EVA index [31]. Furthermore, the EVA index has been calculated using a Spanish-specific population, and unless the sociodemographic characteristics and cardiovascular risk profile are similar to those of our study population, they cannot be compared with other EVA indices used to date [32]. In this study, we included only some parameters associated with the EVA index. Recent studies have expanded the EVA concept to include other endothelial functions [33], such as low mediated dilatation, IMT and inflammatory marker variables (e.g., CRP and interleukin-6) [34]. Future studies should investigate the influence of these elements on the factorial structure of the EVA index.

## Conclusions

Our findings confirm that a single-factor model underlies the EVA index and support that this index accurately classifies HVA and EVA individuals. Overall, EVA index assessment and classification can be useful for clinicians in identifying individuals at risk of developing CVD at an early stage, assessing their risk, and tailoring treatment strategies. Furthermore, the EVA index can provide valuable insights into the underlying mechanisms of CVD and help identify new biomarkers, targeted treatments, and personalized medicine strategies. This can advance research efforts in the field and ultimately lead to better patient outcomes.

### Supplementary Information

**Additional file 1: Table S1.** Inclusion and exclusion criteria for study subjects. **Table S2.** Subject assignments to the two groups (HVA and EVA) using K-means and hierarchical methods. **Table S3.** Silhouette index and the average distance values by sex. **Figure S1.** Dendrogram for the hierarchical clustering analysis. **Figure S2.** Factor loading and goodness-of-fit indexes of single-factor models for EVA index (women). **Figure S3.** Factor loading and goodness-of-fit indexes of single-factor models for EVA index (men). **Figure S4.** Visual representation of the assignment in the two groups (HVA and EVA) in a three-dimensional space using K-means for women. **Figure S5.** Visual representation of the assignment in the two groups (HVA and EVA) in a three-dimensional space using K-means for men. **Figure S6.** Contribution of each variable of the EVA index model to each principal component for women. **Figure S7.** Contribution of each variable of the EVA index model to each principal component for men.

## Data Availability

All data generated or analysed during this study are included in this published article and its Additional files.

## Abbreviations

AGEs: Advanced glycation end products
AIx: Augmentation index
a-PWv: Aortic pulse wave velocity
ARI: Adjusted rand index
CAVI: Cardio ankle vascular index
CFI: Comparative fit index
CRP: Ultrasensitive C-reactive protein
CVD: Cardiovascular disease
DBP: Diastolic blood pressure
EVA: Early vascular ageing
HbA1c: Glycated hemoglobin A1c
HVA: Healthy vascular aging
IMT: Intima media thickness
PCA: Principal component analysis
PP: Pulse pressure
SAF: Skin autofluorescence
SBP: Systolic blood pressure
SRMR: Root mean square residual
STROBE: Strengthening the Reporting of Observational Studies in Epidemiology

## References

[1] Nilsson PM, Boutouyrie P, Laurent S (2009). Vascular ageing: a tale of EVA and ADAM in cardiovascular risk assessment and prevention. Hypertension.

[2] Laurent S (2012). Defining vascular ageing and cardiovascular risk. J Hypertens.

[3] Nilsson PM (2008). Early vascular ageing (EVA): consequences and prevention. Vasc Health Risk Manag.

[4] Glavic MM, Blagus L, Bosnjak V, Frkanec S, Katic L, Domislovic V (2021). Characteristics of healthy vascular ageing (HVA) and early vascular ageing (EVA) in general population. Eh-Uh study (Croatian scientific foundation). J Hypertens.

[5] Cunha PG, Cotter J, Oliveira P, Vila I, Boutouyrie P, Laurent S (2015). Pulse wave velocity distribution in a cohort study: from arterial stiffness to early vascular ageing. J Hypertens.

[6] Currie G, Delles C (2017). Healthy vascular aging. Hypertension.

[7] Berger S, Jordan CO, Lloyd-Jones D, Blumenthal RS (2010). Screening for cardiovascular risk in asymptomatic patients. J Am Coll Cardiol.

[8] D'Agostino RB (2008). General cardiovascular risk profile for use in primary care. The Framingham heart study. Circulation.

[9] Lacey B, Herrington WG, Preiss D, Lewington S, Armitage J (2017). The role of emerging risk factors in cardiovascular outcomes. Curr Atheroscler Rep.

[10] von Elm E, Altman DG, Egger M, Pocock SJ, Gøtzsche PC, Vandenbroucke JP, STROBE Initiative (2014). The strengthening the reporting of observational studies in epidemiology (STROBE) statement: guidelines for reporting observational studies. Int J Surg.

[11] Gomez-Sanchez M, Gomez-Sanchez L, Patino-Alonso MC, Cunha PG, Recio-Rodriguez JI, Alonso-Dominguez R, EVA Investigators (2020). Vascular aging and its relationship with lifestyles and other risk factors in the general Spanish population: early vascular ageing study. J Hypertens.

[12] World Medical Association (2013). World Medical Association Declaration of Helsinki: ethical principles for medical research involving human subjects. JAMA.

[13] Hu L, Bentler PM (1999). Cutoff criteria for fit indexes in covariance structure analysis: conventional criteria versus new alternatives. Struct Equ Model Multidiscip J.

[14] Calinski T, Harabasz J (1974). A dendrite method for cluster analysis. Commun Stat Theory Methods.

[15] Davies DL, Bouldin DW (1979). A cluster separation measure. TPAMI.

[16] Rousseeuw PJ (1987). Silhouettes: a graphical aid to the interpretation and validation of cluster analysis. J Comput Appl Math.

[17] Arthur D, Vassilvitskii S. K-means++: the advantages of careful seeding. In: ACM-SIAM. 2007. p. 1027–35.

[18] Murtagh F, Legendre P (2014). Ward’s hierarchical agglomerative clustering method: which algorithms implement Ward’s criterion?. J Classif.

[19] Gates AJ, Ahn YY (2017). The impact of random models on clustering similarity. JMLR.

[20] Jolliffe IT, Cadima J (2016). Principal component analysis: a review and recent developments. Philos Trans R Soc.

[21] Nilsson PM, Boutouyrie P, Cunha P, Kotsis V, Narkiewicz K, Parati G (2013). Early vascular ageing in translation: from laboratory investigations to clinical applications in cardiovascular prevention. J Hypertens.

[22] den Dekker MA, Zwiers M, van den Heuvel ER, de Vos LC, Smit AJ, Zeebregts CJ (2013). Skin autofluorescence, a non-invasive marker for AGE accumulation, is associated with the degree of atherosclerosis. PLoS ONE.

[23] Katakami N, Osonoi T, Takahara M (2015). Clinical utility of glycated albumin and glycated hemoglobin for the detection of early diabetic vascular complications. Diabetes Res Clini Pract.

[24] Laurent S, Boutouyrie P (2015). The structural factor of hypertension: large and small artery alterations. Circ Res.

[25] Seals DR, Jablonski KL, Donato AJ (2011). Ageing and vascular endothelial function in humans. Clin Sci (Lond).

[26] Forbes JM, Cooper ME (2013). Mechanisms of diabetic complications. Physiol Rev.

[27] Meerwaldt R, Graaff R, Oomen PH, Links TP, Jager JJ, Alderson NL (2004). Simple non-invasive assessment of advanced glycation endproduct accumulation. Diabetologia.

[28] Basta G, Schmidt AM, De Caterina R (2004). Advanced glycation end products and vascular inflammation: implications for accelerated atherosclerosis in diabetes. Cardiovasc Res.

[29] Karrasch T, Brüske I, Gieger C, EVA Study (2017). What is healthy vascular ageing (EVA)? Different definitions and their impact on research and interpretation. Eur J Prev Cardiol.

[30] Wang M, Monticone RE, Lakatta EG (2010). Arterial ageing: a journey into subclinical arterial disease. Curr Opin Nephrol Hypertens.

[31] Rothman KJ, Greenland S (2005). Causation and causal inference in epidemiology. Am J Public Health.

[32] Rothman KJ, Gallacher JE, Hatch EE (2013). Why representativeness should be avoided. Int J Epidemiol.

[33] Donato AJ, Eskurza I, Silver AE, Silver AE, Levy AS, Pierce GL (2007). Direct evidence of endothelial oxidative stress with ageing in humans: relation to impaired endothelium-dependent dilation and upregulation of nuclear factor-kappaB. Circ Res.

[34] Donato AJ, Morgan RG, Walker AE, Lesniewski LA (2015). Cellular and molecular biology of ageing endothelial cells. JMCC.

