# Supplementary material for: Early vascular aging as an index of cardiovascular risk in healthy adults: confirmatory factor analysis from the EVasCu study
Source: Cardiovasc Diabetol. 2023 Aug 17;22:209. doi: 10.1186/s12933-023-01947-9 (PMC10436435; doi:10.1186/s12933-023-01947-9)

Additional file 1

Table S1. Inclusion and exclusion criteria for study subjects.

Inclusion criteria	Exclusion criteria
Healthy subjects	Participation in another study
Age >18 years	Diagnosed pathologies
Clinically stable for 6 weeks before the study	Under pharmacological treatment
Written informed consent	

Table S2. Subject assignments to the two groups (HVA and EVA) using K-means and hierarchical methods.

		Hierarchical	
		Healthy Vascular Aging	Early Vascular Aging
K-means	Healthy Vascular Aging	224	15
	Early Vascular Aging	0	142

Table S3. Silhouette index and the average distance values by sex.

	Men	Women
Silhouette index	0,6113	0,6677
Average distance	47,602	44,248

Figure S1. Dendrogram for the hierarchical clustering analysis.

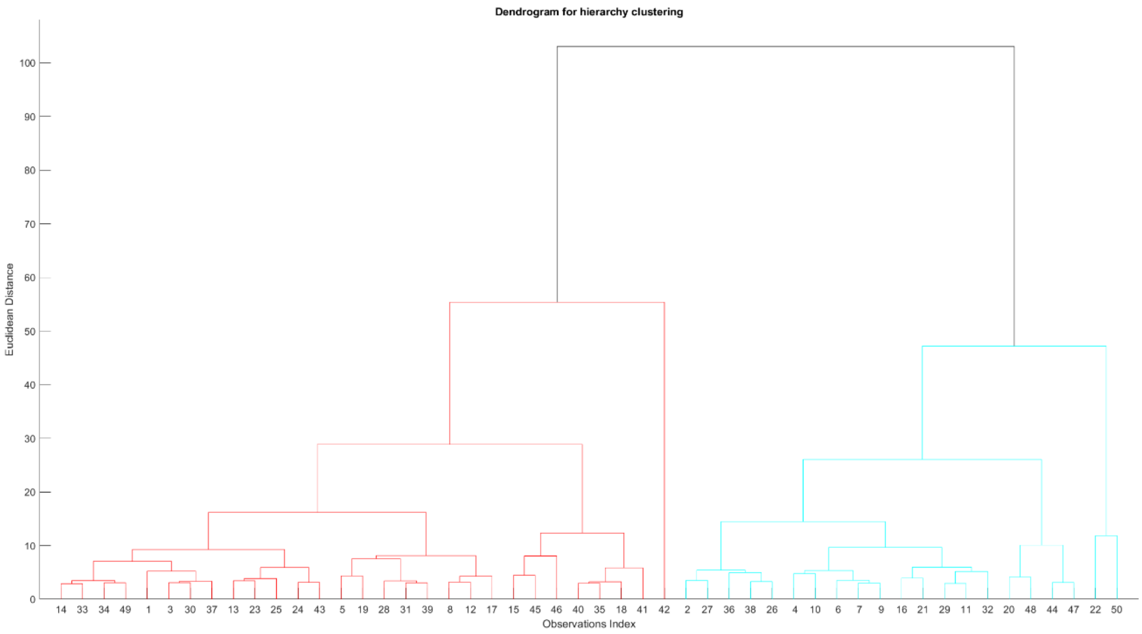

Figure S2. Factor loading and goodness-of-fit indexes of single-factor models for EVA index (women).

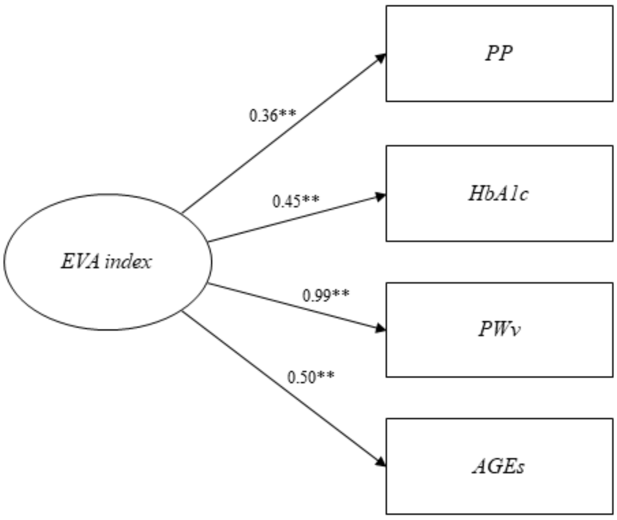

Chi2 (df) = 1.05 (2), df = 2, p = 0.591, CFI = 1.000, SRMR = 0.017
**Indicates p<0.010

Figure S3. Factor loading and goodness-of-fit indexes of single-factor models for EVA index (men).

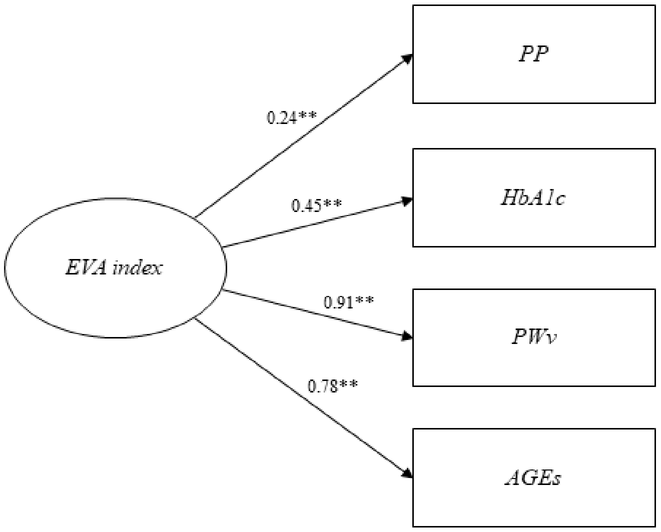

Chi2 (df) = 0.41 (2), p = 0.816, CFI = 1.000, SRMR = 0.011
**Indicates p<0.010

Figure S4. Visual representation of the assignment in the two groups (HVA and EVA) in a three-dimensional space using K-means for women.

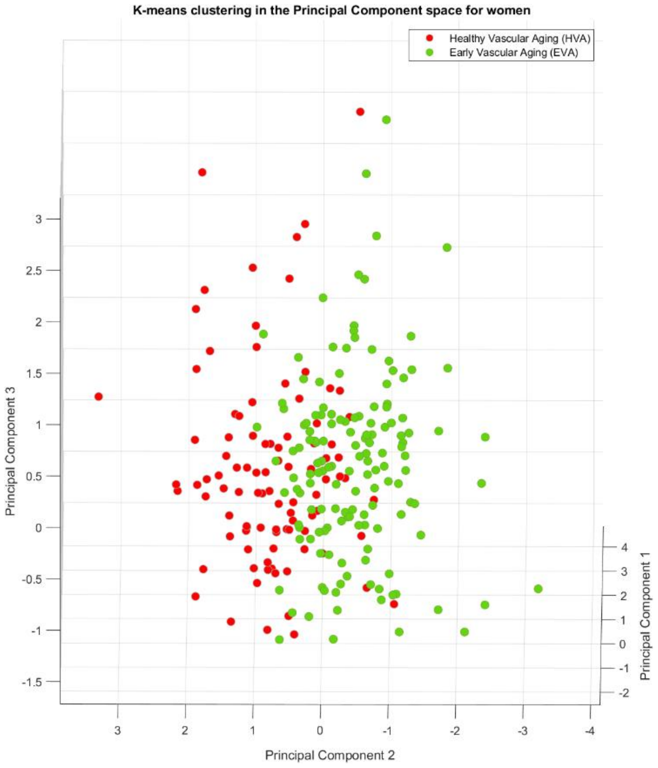

Figure S5. Visual representation of the assignment in the two groups (HVA and EVA) in a three-dimensional space using K-means for men.

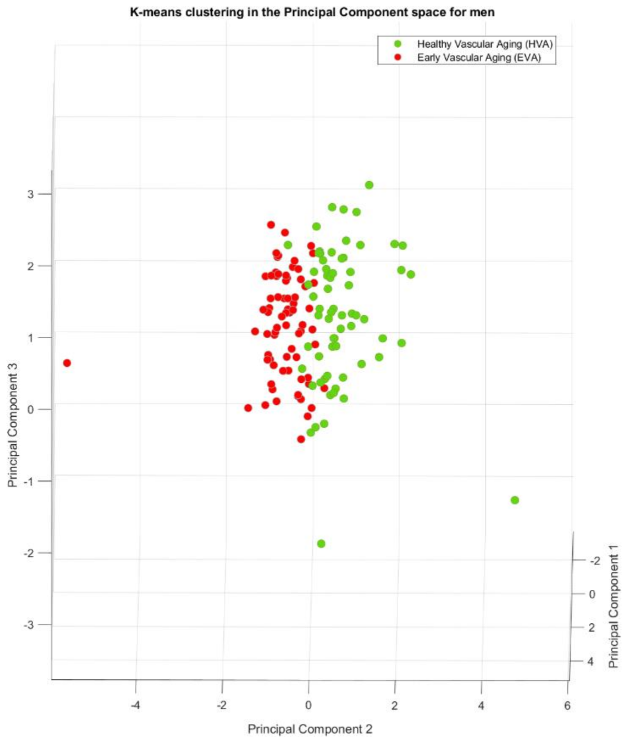

Figure S6. Contribution of each variable of the EVA index model to each principal component for women.

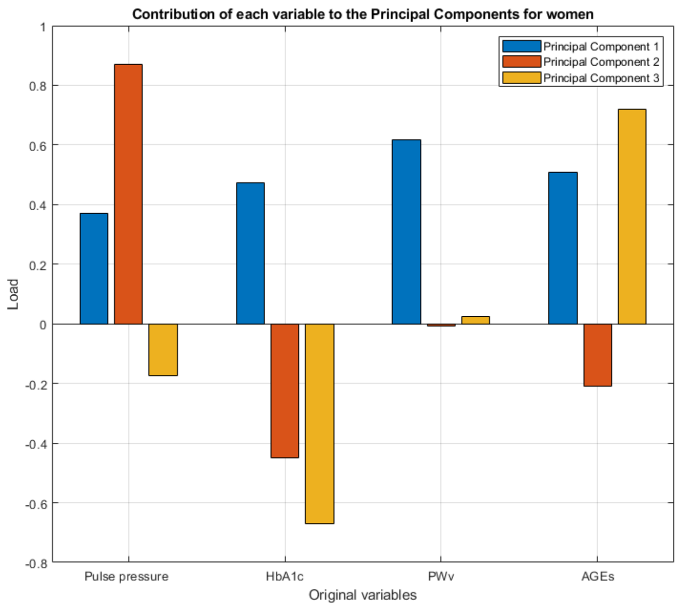

Figure S7. Contribution of each variable of the EVA index model to each principal component for men.

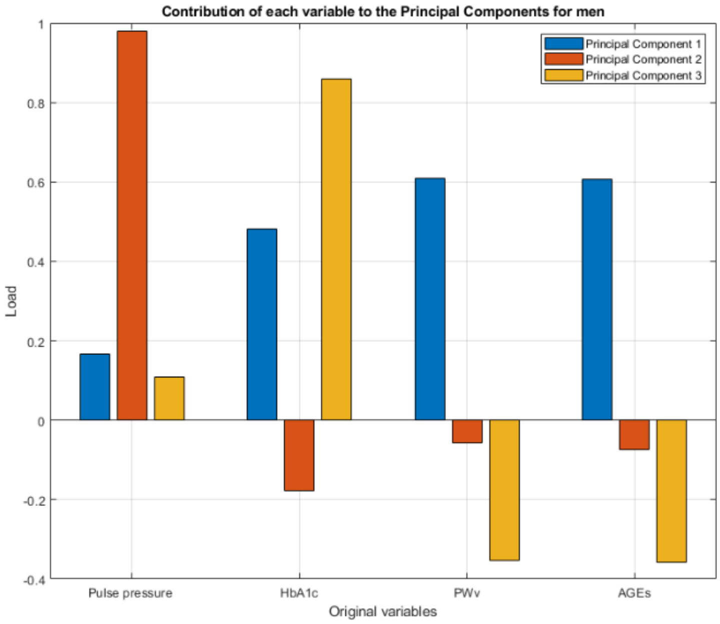

Supplement: Supplementary file 1 — Additional file 1: Table S1. Inclusion and exclusion criteria for study subjects. Table S2. Subject assignments to the two groups (HVA and EVA) using K-means and hierarchical methods. Table S3. Silhouette index and the average distance values by sex. Figure S1. Dendrogram for the hierarchical clustering analysis. Figure S2. Factor loading and goodness-of-fit indexes of single-factor models for EVA index (women). Figure S3. Factor loading and goodness-of-fit indexes of single-factor models for EVA index (men). Figure S4. Visual representation of the assignment in the two groups (HVA and EVA) in a three-dimensional space using K-means for women. Figure S5. Visual representation of the assignment in the two groups (HVA and EVA) in a three-dimensional space using K-means for men. Figure S6. Contribution of each variable of the EVA index model to each principal component for women. Figure S7. Contribution of each variable of the EVA index model to each principal component for men. [file 12933_2023_1947_MOESM1_ESM.pdf]
